# 

**Published:** 1888-04

**Authors:** 


### CONTENTS.

### COMMUNICATIONS.

President's Address........................................................ 161
My Experience with the Dental Pulp........................................ 164
Intermittent Pressure.—Its Relation to Orthodontia..................... 169
The Dental Pulp.............................................................. 175
Conservation of the Dental Pulp............................................ 177
Gangrenous Tooth Pulps as Centers of Infection......................... 180
Remarks on Tin and Gold or "Tg"........................................... 186
Non-Malignant and Malignant Tumors........................................ 192

### SELECTIONS.

Antiseptics in the Mouth.................................................... 194
Photography in Surgery...................................................... 201
How to Clean the Hands...................................................... 204
Chloroform and the Constant Current in Neuralgia........................ 205
SALMAGUNDI.................................................................... 205

### EDITORIAL.

American Dental Association................................................ 208
American Medical Association............................................... 209
Dental College of the University of Michigan............................. 210
Dental Metallurgy............................................................ 210
Dentist's Manual of Special Chemistry..................................... 211
The Next Meeting of the Association of American Medical Editors 212

